# Urine concentrations of selected trace metals in a cohort of Irish adults

**DOI:** 10.1007/s11356-022-21169-y

**Published:** 2022-06-02

**Authors:** James P. K. Rooney, Bernhard Michalke, Gráinne Geoghegan, Mark Heverin, Stephan Bose-O’Reilly, Orla Hardiman, Stefan Rakete

**Affiliations:** 1 Institute and Clinic for Occupational-, Social- and Environmental Medicine, University Hospital, LMU Munich, Munich, Germany; 2 Academic Unit of Neurology, Trinity Biomedical Sciences Institute, Trinity College Dublin, Dublin, Ireland; 3 Research Unit Analytical BioGeoChemistry, Helmholtz Center Munich, German Research Center for Environmental Health, Neuherberg, Germany; 4 Institute of Public Health, Medical Decision Making and Health Technology Assessment, Department of Public Health, Medical Informatics and Technology, Health Services Research and Health Technology Assessment, UMIT - Private University for Health Sciences, Hall in Tirol, Austria; 5 Department of Neurology, Beaumont Hospital, Glasnevin, Dublin, Ireland

**Keywords:** Metals, Biomonitoring, Cohort study, Ireland

## Abstract

Human biomonitoring studies are of increasing importance in regulatory toxicology; however, there is a paucity of human biomonitoring data for the Irish population. In this study, we provide new data for urinary biomarker concentrations of aluminium, arsenic, cadmium, chromium, copper, mercury, manganese, lead and selenium. One hundred urine samples, collected between 2011 and 2014 from healthy participants of the EuroMOTOR project, were randomly selected. Metal concentrations were measured via ICPMS. Descriptive statistics for each of the metals stratified by gender were performed. There were 58 male and 42 female participants and metals were detectable for all samples. Geometric mean urinary concentrations for each metal in males were as follows: aluminium 8.5 μg/L, arsenic 8.1 μg/L, cadmium 0.3 μg/L, chromium 0.5 μg/L, copper 5.1 μg/L, mercury 0.4 μg/L, manganese 0.3 μg/L, lead 1.3 μg/L and selenium 10.8 μg/L; and in females: aluminium 8.5 μg/L, arsenic 10.2 μg/L, cadmium 0.4 μg/L, chromium 0.6 μg/L, copper 5.6 μg/L, mercury 0.3 μg/L, manganese 0.2 μg/L, lead 1.6 μg/L and selenium 13.7 μg/L. We observed higher geometric mean concentrations in women for arsenic, cadmium, chromium, copper, lead and selenium, with equal geometric mean concentrations for aluminium and manganese, leaving only mercury with lower geometric mean concentrations in women. Aluminium, cadmium, chromium, lead and urinary concentrations of metals were slightly elevated compared to European data, while for arsenic, copper, manganese and selenium, Irish levels were lower. Our findings highlight that there are differences in urinary metal concentrations between European populations.

**Supplementary Information:**

The online version contains supplementary material available at 10.1007/s11356-022-21169-y.

## Introduction

Human biomonitoring studies are of increasing importance in modern regulatory toxicology, providing a means to measure population exposures to chemicals and investigate associations between biomarkers of these chemicals and human health outcomes. Internationally, several long-running human biomonitoring programmes are well established including NHANES in the USA, and the Korean NHANES programme. In Europe in 2009, the COPHES project (http://www.eu-hbm.info/cophes) developed harmonised protocols allowing the collection of comparable HBM data throughout Europe. These were then piloted in its twin project, the DEMOCOPHES project (http://www.eu-hbm.info/democophes), between 2010 and 2012. These were succeeded by the HBM4EU project (https://www.hbm4eu.eu) which runs from 2017 to 2022. In Ireland, however, information on biological markers of metal exposures is lacking.

The most important study to measure toxic metals in Irish individuals previously was the DEMOCOPHES study. This was an EU wide study of human biomonitoring amongst children aged 6 to 11 and mothers aged 45 or younger in Europe that aimed to develop common strategies and scientific protocols across member states using 4 exposures as a pilot: urinary cadmium, hair mercury, urinary cotinine and urinary phthalate metabolites (Schindler et al. 2014). Irish mothers who smoked (*N* = 35) had median urinary cadmium concentration of 0.38 µg/g creatinine, non-smoking mothers (*N* = 82) had median urinary cadmium concentrations of 0.28 µg/g creatinine, and children (*N* = 119) had median urinary cadmium concentrations below the limit of quantification (LOQ) (Berglund et al. 2015). Hair from Irish mothers (*N* = 120) had median mercury concentration of 0.188 μg/g, while Irish children (*N* = 120) had median mercury concentration of 0.100 μg/g. Few other studies of toxic metal biomarkers from the Irish population exist. A 1998 study sought to determine reference levels for urinary antimony in Irish infants (Cullen et al. 1998). A sample of 100 Irish infants was recruited from the Eastern area birth register and a median urinary antimony concentration of 0.42 μg/g creatinine with a 95th percentile of 2.6 ng/mg creatinine was determined (Cullen et al. 1998).

In the current study, we provide new data regarding urinary biomarker concentrations amongst Irish adults for the following metallic minerals: copper and selenium; trace minerals: chromium and manganese and toxins: aluminium, arsenic, cadmium, mercury and lead.

## Methods

### Study population

The EuroMOTOR project was an international case–control study investigating the role of environmental exposures in ALS that included amyotrophic lateral sclerosis (ALS) patients and age and sex-matched controls (D’Ovidio et al. 2017). ALS patients had been recruited from the population-based Irish ALS register between 2011 and 2014, with controls recruited via individual matching to patients by gender, age (± 5 years) and by location (controls were recruited through general practitioners located within the same county as matched patients) (D’Ovidio et al. 2017). As part of the EuroMOTOR study design, controls with a history of neurological conditions were excluded. A sample of 100 controls was randomly selected from 305 controls who had provided urine samples. Corresponding age, gender, BMI and education data were extracted from the EuroMOTOR dataset.

### Sample collection

Urine samples were collected from participants at the time of interview for the EuroMOTOR survey (D’Ovidio et al. 2017). Urine samples were collected in 10-ml sterile collection tubes with no preservative. Urine samples were centrifuged immediately at 1500 g for 15 min. The supernatant was then aliquoted in 2-ml cryovials, as 4 × 2 ml aliquots and transported to the laboratory at 4 °C. Within 24 h, samples were stored at − 80 °C.

### Element determination by ICP-sf-MS measurement

Urine samples were thawed slowly and diluted 1/5 with 0.5% HNO_3_. ^103^Rh was administered to each sample at a final concentration of 1 µg/L as internal standard and subsequently analysed. An ELEMENT 2, ICP-sf-MS instrument (Thermo Scientific, Bremen, Germany) was employed for the determination of ^112^Cd, ^202^Hg and ^208^Pb in low-resolution mode, ^27^Al, ^52^Cr, ^55^Mn and ^65^Cu in medium-resolution mode, whereas ^75^As and ^77^Se were measured in high-resolution mode. Sample introduction was carried out using an ESI-Fast-system (Elemental Scientific, Mainz, Germany) connected to a Micromist nebuliser with a cyclon spray chamber. The RF power was set to 1200 W, the plasma gas was 15 L Ar /min, whereas the nebuliser gas was approximately 0.9 L Ar/min after daily optimization. The limits of detection (LOD) for each metal in non-diluted urine were as follows: Al 3 ng/L, As 8 ng/L, Cd 1.5 ng/L, Cr 1.5 ng/L, Cu 1.5 ng/L, Hg 1.5 ng/L, Mn 1.5 ng/L, Pb 1.5 ng/L, Se 16 ng/L.

### Quality control for element determinations

The determination methods had been validated previously by successful regular laboratory intercomparison studies. Routinely, each ten measurements three blank determinations and a control determination of a certified control standard for all mentioned elements were performed. Calculation of results was carried out on a computerized lab-data management system, relating the sample measurements to calibration curves, blank determinations and control standards.

### Statistical analysis

Demographic variables collected included age and sex, maximum education level, smoking status (ever vs never) and BMI. Geometric and arithmetic means and percentiles (P10, P25, P50, P75, P90, P95) were calculated for the full cohort and stratified by sub-groups. Where case counts in a given category were < 5, data was censored for privacy reasons. All statistical analyses were carried out using R Statistical Software version 4.0.5 (R Core Team 2022) and additional packages (Taiyun and Simko 2021; Wickham 2017; Yoshida and Bartel 2021). (Analysis code is available at: https://github.com/jpkrooney/Urinary_Metals_Irish_Adults.)

## Results

**Fig. 1:**
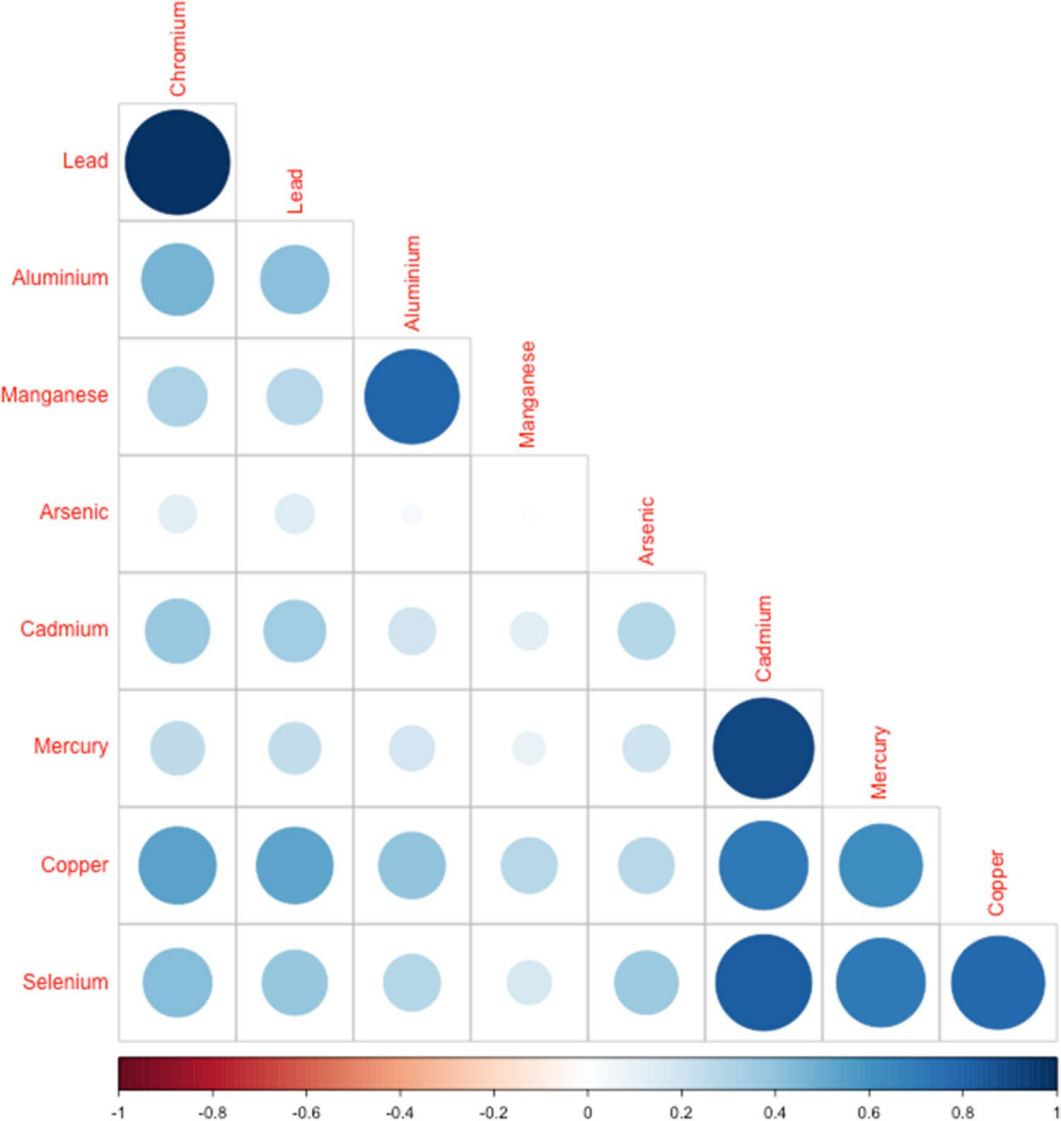
Correlation matrix of urinary toxic metal concentrations adjusted for creatinine

There were 58 male and 42 female participants. Demographic details are shown in Table 1. Demographic characteristics were similar across genders, although men had a higher mean BMI at 27.3 compared to 26.5 in women, and a greater percentage of women (45%) had post-secondary education compared to men (39%). Urinary metal concentrations from the 100 participants are summarised in Table 2. (Supplementary Table 1 displays the urinary metal concentrations adjusted for urinary creatinine concentration). Detection rates were 100% for all metals. For arsenic, cadmium, chromium, copper, lead and selenium, the geometric mean concentration was marginally higher in women than in men (Table 2). These differences were reflected also in the percentile figures, particularly at the higher percentiles (Table 2). The concentrations were only higher in men across all percentiles for total mercury concentration (Table 2). Similar gender related patterns are observed here with creatinine adjusted metal concentrations being higher in woman compared to men for all metals, likely reflecting that creatinine concentration is lower in women in the 25th to 75th percentile range (Supplementary Table 1). Figure 1 displays the correlations between metal concentrations in the samples after adjustment for creatinine. Most of the metals were positively correlated with each other, with only arsenic appearing to be uncorrelated with other metals. Cadmium, copper, mercury and selenium concentrations were particularly strongly positively correlated with each other. Table 3 summarises data geometric means and 95th percentiles from the current study in context with data from other recent studies. For 12 samples, urinary cadmium concentrations were found to be above the age -specific HBM1 threshold recently defined by the HBM4EU project for urinary cadmium (Lamkarkach et al. 2021; Schulz et al. 2011). Only 2 samples were above the HBM1 threshold for mercury by the German Human Biomonitoring Commission. Threshold levels have not been defined for the other metals measured.

**Table 1:** Demographic details by gender for 100 Irish adults

	Female	Male
N	42	58
Age, mean (sd)	66.5 (11.2)	64.1 (11.0)
BMI, mean (sd)	26.5 (6.1)	27.3 (3.8)
Education		
Primary	8 (19%)	16 (28%)
Secondary	15 (36%)	19 (33%)
Technical	10 (24%)	9 (15%)
University	9 (21%)	14 (24%)

**Table 2:** Means and percentiles of urinary toxic metal concentrations by gender in 100 Irish individuals

Toxic metal	Sex	N	Arithmetic mean (μg/L)	Geometric mean (μg/L)	P10% (μg/L)	P25% (μg/L)	P50% (μg/L)	P75% (μg/L)	P90% (μg/L)	P95% (μg/L)
Aluminium	Female	42	17.0	8.5	2.6	5.0	7.7	13.2	31.6	53.3
	Male	58	13.7	8.5	1.9	5.8	10.2	15.9	26.2	40.3
Arsenic	Female	42	37.9	10.2	1.4	3.5	9.8	24.9	71.2	223.2
	Male	58	24.8	8.1	1.3	2.6	7.6	22.2	58.7	69.4
Cadmium	Female	42	1.0	0.4	0.1	0.2	0.5	0.9	1.5	1.8
	Male	58	0.4	0.3	0.1	0.2	0.3	0.6	0.8	0.9
Chromium	Female	42	1.1	0.6	0.1	0.5	0.8	1.0	1.2	1.5
	Male	58	0.7	0.5	0.1	0.5	0.8	0.9	1.0	1.1
Copper	Female	42	12.0	5.6	1.0	3.2	6.2	11.8	20.4	27.1
	Male	58	7.7	5.1	1.0	3.1	7.3	11.5	14.6	18.6
Mercury	Female	42	3.6	0.3	0.0	0.1	0.4	0.8	1.5	2.1
	Male	58	0.9	0.4	0.1	0.1	0.6	1.0	2.2	2.5
Manganese	Female	42	0.6	0.2	0.0	0.1	0.2	0.4	0.9	1.6
	Male	58	0.3	0.2	0.0	0.1	0.2	0.4	0.8	0.9
Lead	Female	42	2.6	1.6	0.2	1.3	2.4	2.8	3.9	4.2
	Male	58	1.9	1.3	0.2	1.1	2.2	2.4	2.8	3.0
Selenium	Female	42	29.2	13.7	2.6	7.7	15.5	30.5	49.2	53.9
	Male	58	17.2	10.8	2.1	4.9	13.7	28.9	37.3	39.7

**Table 3:** Reference values (μg/L) for trace elements from international studies

	Study reference	Country		Study period	Sex	Age range	*N*	GM	P95%
Al		**Ireland**		**2011–2014**	**Female**	**40–88**	**42**	**8.5**	**53.3**
					**Male**	**37–84**	**58**	**8.5**	**40.3**
	(Nisse et al. 2017)	Northern France		2008**–**2010	Female	20**–**59	968	2.28	12.7
					Male		942	1.53	9.94
	(Hoet et al. 2013)	Belgium		2010**–**2011	Mixed	18**–**80	1001	2.15	9.27
As		**Ireland**		**2011–2014**	**Female**	**40–88**	**42**	**10.2**	**223.2**
					**Male**	**37–84**	**58**	**8.1**	**69.4**
	(Nisse et al. 2017)	Northern France		2008**–**2010	Female	20**–**59	968	18.2	127
					Male		942	19.2	136
	(Hoet et al. 2013)	Belgium		2010**–**2011	Mixed	18**–**80	1001	15.4	157
	(CDC, 2019)	USA		2015**–**2016	Mixed	> 20	1794	N/A	49.9
	(Saravanabhavan et al. 2017)	Canada		2009**–**2011	Mixed	3**–**79	2480	N/A	27
Cd		**Ireland**		**2011–2014**	**Female**	**40–88**	**42**	**0.4**	**1.8**
					**Male**	**37–84**	**58**	**0.3**	**0.9**
	(Berglund et al. 2015)	Ireland		2011**–**2012	Female (smokers)	24**–**52	35	0.33^b^	1.07^b^
					Female (non-smokers)		82	0.24^b^	0.63^b^
	(Nisse et al. 2017)	Northern France		2008**–**2010	Female	20**–**59	968	0.39	1.3
					Male		942	0.37	1.36
	(Vogel et al. 2021)	Germany		2015**–**2017	Female	3**–**17	1092	0.071	0.23
					Male		1158	0.074	0.26
	(Hoet et al. 2013)	Belgium		2010**–**2011	Mixed	18**–**80	1001	0.228	1.06
	(Batáriová et al. 2006)	Czech Republic		2002**–**2003	Female	18**–**58	160	0.33	1.48
					Male		497	0.27	1.24
	(CDC, 2019)	USA		2015**–**2016	Mixed	> 20	1794	N/A	1.08
	(Saravanabhavan et al. 2017)	Canada		2009**–**2011	Mixed	20**–**79	1196	N/A	1.3
					Mixed (never smokers)	> 18	613	0.31	N/A
	(Sun et al. 2016)	China		2013–2014	Mixed (former smokers)	> 18	46	0.41	N/A
					Mixed (current smokers)	> 18	237	0.44	N/A
Cr		**Ireland**		**2011–2014**	**Female**	**40–88**	**42**	**0.6**	**1.5**
					**Male**	**37–84**	**58**	**0.5**	**1.1**
	(Nisse et al. 2017)	Northern France		2008**–**2010	Female	20**–**59	968	0.39	1.62
					Male		942	0.39	1.54
	(Vogel et al. 2021)	Germany		2015**–**2017	Female	3**–**17	1158	0.4	0.84
					Male		1092	0.386	0.8
	(Hoet et al. 2013)	Belgium		2010**–**2011	Mixed	18**–**80	1001	0.103	0.45
Cu		**Ireland**		**2011–2014**	**Female**	**40 –88**	**42**	**5.6**	**27.1**
					**Male**	**37–84**	**58**	**5.1**	**18.6**
	(Hoet et al. 2013)	Belgium		2010**–**2011	Mixed	18 **–**80	1001	6.94	19.6
	(Saravanabhavan et al. 2017)	Canada		2009**–**2011	Mixed	20**–**79	1513	N/A	25
Hg		**Ireland**		**2011–2014**	**Female**	**40–88**	**42**	**0.3**	**2.1**
					**Male**	**37–84**	**58**	**0.4**	**2.5**
	(Nisse et al. 2017)	Northern France		2008**–**2010	Female	20**–**59	968	0.82	6.31
					Male		942	0.92	6.84
	(Vogel et al. 2021)	Germany		2015**–**2017	Female	3**–**17	1089	0.062	0.23
					Male		1153	0.072	0.28
	(Hoet et al. 2013)	Belgium		2010**–**2011	Mixed	18**–**80	1001	0.26	1.88
	(Batáriová et al. 2006)	Czech Republic		2002**–**2003	Female	18**–**58	160	0.96	11.8
					Male		497	0.52	5.35
	(Castaño et al. 2019)	Spain		2009**–**2010	Female	18**–**50	832	1.14	3.99
					Male		872	1.09	4.23
	(CDC, 2019)	USA		2015**–**2016	Mixed	> 20	1802	N/A	1.22
	(Saravanabhavan et al. 2017)	Canada		2012**–**2013	Female	20**–**79	241	N/A	N/A
					Male		217	N/A	0.73
Mn		**Ireland**		**2011–2014**	**Female**	**40–88**	**42**	**0.2**	**1.6**
					**Male**	**37–84**	**58**	**0.2**	**0.9**
	(Nisse et al. 2017)	Northern France		2008**–**2010	Female	20**–**59	968	0.29	1.06
					Male		942	0.27	1.07
	(Hoet et al. 2013)	Belgium		2010**–**2011	Mixed	18—80	1001	N/A	0.355
	(CDC, 2019)	USA		2015**–**2016	Mixed	> 20	1794	N/A	0.28
Pb			**Ireland**	**2011–2014**	**Female**	**40–88**	**42**	**1.6**	**4.2**
					**Male**	**37–84**	**58**	**1.3**	**3.0**
	(Nisse et al. 2017)		Northern France	2008**–**2010	Female	20**–**59	968	0.9	3.24
					Male		942	1.26	4.26
	(Hoet et al. 2013)		Belgium	2010**–**2011	Mixed	18**–**80	1001	0.74	2.81
	(CDC, 2019)		USA	2015**–**2016	Mixed	> 20	1794	N/A	1.38
	(Saravanabhavan et al. 2017)		Canada	2015**–**2016	Mixed	20**–**79	3210	N/A	1.9
Se			**Ireland**	**2011–2014**	**Female**	**40–88**	**42**	**13.7**	**53.9**
					**Male**	**37–84**	**58**	**10.8**	**39.7**
	(Hoet et al. 2013)		Belgium	2010**–**2011	Mixed	18**–**80	1001	21.6	61.6
	(Saravanabhavan et al. 2017)		Canada	2009**–**2011	Mixed	20**–**79	3211	N/A	120

Geometric mean (GM) and 95.^th^ percentile (P95%) values given as μg/L unless otherwise stated. Aluminium (Al), Arsenic (As), Cadmium (Cd), Chromium (Cr), Copper (Cu), Mercury (Hg), Manganese (Mn), Lead (Pb), Selenium (Se)

^a^95.^th^ percentile values not calculated for Ireland due to sample size

^b^Values given as μg/g creatinine

## Discussion

In this study, we have described the concentrations of 9 metals in urine samples from 100 Irish adults recruited between 2011 and 2014. Previous comparable studies in this population are rare; however, our finding of geometric mean urinary cadmium concentration in women of 0.7 μg/g creatinine is higher than that measured in Irish mothers as part of the DEMOCOPHES study where non-smoking mothers (*N* = 82) had a geometric mean of 0.24 μg/g creatinine and smoking mothers (*N* = 35) had a geometric mean of 0.33 μg/g creatinine (Berglund et al. 2015) (Table 3). We note however, that while the DEMOCOPHES participants were recruited between 2011 and 2012 which overlapped the recruitment period of our study, the mean age of our female participants was 66.5, while DEMOCOPHES recruited women between the ages of 24 to 52(Berglund et al. 2015). It is known that cadmium accumulates in the kidney and increasing in urinary cadmium with age has been observed in Chinese (Sun et al. 2016) and Swiss (Jenny-Burri et al. 2015) populations. Furthermore, we found unadjusted urinary cadmium concentrations in women (GM: 0.4 μg/L) to be comparable to those found in 968 French women (GM: 0.39 μg/L) (Nisse et al. 2017) and 160 Czech women (GW: 0.33 μg/L) (Batáriová et al. 2006), somewhat higher than those found in a cohort of 1001 mixed gender Belgians (GM: 0.228 μg/L) (Hoet et al. 2013), but notably higher than that found in 1092 German women (GM: 0.071 μg/L) (Vogel et al. 2021) (Table 3). In men, we found a geometric mean unadjusted urinary cadmium concentration of 0.3 μg/L which is comparable to 942 French men (GM: 0.37 μg/L) (Nisse et al. 2017), somewhat higher than concentrations in 497 Czech men (GM: 0.27 μg/L) (Batáriová et al. 2006), but again, concentrations were markedly lower in 1158 German men (GM: 0.074 μg/L) (Vogel et al. 2021) (Table 3). For mixed genders at the 95th percentile, concentrations in the USA (1.08 μg/L)(CDC, 2019) and Canada (1.3 μg/L) (Saravanabhavan et al. 2017) were higher than that in Irish males (0.9 μg/L) but not females(1.8 μg/L); however, we interpret this cautiously due to our small sample size.

For other metals, we could not find comparable Irish data. However, European/International data is available. Our finding of aluminium concentration GM: 8.5 μg/L in both men and women was higher than urinary concentrations in French women (GM: 2.28 μg/L) and men (GM: 1.53 μg/L) (Nisse et al. 2017), and also higher than that in in a mixed cohort of 1001 Belgian men and women (GM: 2.15 μg/L) (Hoet et al. 2013) (Table 3). For arsenic, we found concentrations of GM: 10.2 μg/L in women and GM: 8.1 μg/L in men. These concentrations were lower than arsenic concentrations found in the in French women (GM: 18.2 μg/L) and men (GM: 19.2 μg/L) (Nisse et al. 2017), and lower than that in Belgians (GM: 15.4 μg/L) (Hoet et al. 2013). For chromium, comparison data was again available for Belgium, France and Germany. Geometric mean concentrations in Irish women (GM: 0.6 μg/L) and men (GM: 0.5 μg/L) were higher than those in the other countries with Belgium having the lowest concentrations (GM: 0.1 μg/L) (Table 3). For copper, urinary concentrations in Irish men (GM: 5.1 μg/L) and women (GM: 5.6 μg/L) were lower than those in a mixed Belgian cohort (GM: 8.18 μg/L) in men and women combined (Hoet et al. 2013).

The only previous study of mercury biomarkers in the Irish population used hair mercury levels and is therefore not directly comparable (Cullen et al. 2014). Nevertheless, comparable data was available from a range of countries (Table 3). Mercury levels in Irish women (GM: 0.3 μg/L) and men (GM: 0.4 μg/L) were higher than those in German women (GM: 0.062 μg/L) and men GM: 0.072 μg/L) (Vogel et al. 2021), and those in the Belgian mixed cohort (GM: 0.26 μg/L) (Hoet et al. 2013). In contrast, Irish geometric mean urinary mercury concentrations were lower when compared to Czech women (GM: 0.96 μg/L) and men (GM: 0.52 μg/L) (Batáriová et al. 2006), French women (GM: 0.82 μg/L) and men (GM: 0.92 μg/L) (Nisse et al. 2017) and in Spanish women (GM: 1.14 μg/L) and men (GM: 1.09 μg/L) (Castaño et al. 2019) than in the Irish data.

Urinary manganese concentrations had a geometric mean of 0.2 μg/L in both Irish men and women, which is lower than that in French women (GM: 0.29 μg/L) and men (GM: 0.27 μg/L) (Nisse et al. 2017). At the 95th percentile, however, Irish levels (women 95th: 1.6 μg/L, men 95th: 0.9 μg/L) were higher than those reported by NHANES (2015–2016 women 95th: 0.35 μg/L, men 95th 0.27 μg/L)(CDC 2019).

Lead levels in Irish women (GM: 1.6 μg/L) were notably high compared to French women (GM: 0.9 μg/L) (Nisse et al. 2017); however, for Irish men (GM: 1.3 μg/L), levels were comparable to that of French men (GM: 1.26 μg/L) (Nisse et al. 2017). The age profile of the French study (20 to 59 years) differs to ours (37 to 88 years); however, this is unlikely to explain gender-specific differences. The mixed gender Belgian cohort had a blood lead level GM = 0.74 μg/L, lower than Irish results for either gender. Finally, for urinary selenium concentrations in Irish women (median: 15.5 μg/L) and men (median: 13.7 μg/L) were lower than the Belgian cohort (GM 21.6 μg/L) (Hoet et al. 2013).

### Strengths and weaknesses

The EuroMOTOR study was a population-based study; however, with just 100 participants, our sample size is not large enough to establish population normative data for urinary metals in the Irish population. In addition, as the matching of EuroMOTOR selected controls with an age-range reflecting the age range of typical ALS incidence, our results do not reflect all ages in the population. Nevertheless, our study provides new data where it was previously almost entirely lacking. Specifically with regard to lead measurements, urinary lead concentration typically reflects short-term exposure. It is therefore less suitable for human biomonitoring than the analysis of lead in blood, where approximately 95% of the lead is bound to the erythrocytes.

## Conclusions

Here, we report the first data on multiple metals in urinary samples collected from Irish adults. We observed slightly higher geometric mean concentrations in women for arsenic, cadmium, chromium, copper, lead and selenium, with equal geometric mean concentrations for aluminium and manganese, leaving only mercury with lower geometric mean concentrations in women. In our cohort, aluminium, cadmium, chromium, lead and urinary concentrations of metals appear comparable but slightly elevated compared to available European data, while for arsenic, copper, manganese and selenium, Irish levels are slightly lower than available European data. For urinary total mercury concentration, Ireland lies in the mid-range of European data. Our findings highlight that there are differences in urinary metal concentrations between European populations. There is a lack of human biomonitoring data for metal concentrations in Ireland, and further explanation of these results will require investigation via purpose-built international human biomonitoring programmes, such as the upcoming EU-funded Partnership for the Assessment of Risk from Chemicals (PARC) (https://www.efsa.europa.eu/en/funding-calls/european-partnership-assessment-risks-chemicals-parc).

## Supplementary Information

Below is the link to the electronic supplementary material.

Supplementary file1 (DOCX 16 KB)

## Data Availability

Anonymised data are available upon reasonable request. Requests should be sent to HARDIMAO@tcd.ie.

## Code availability

Analysis code is  archived at: 10.5281/zenodo.6606623.

## References

[CR1] Batáriová A, Spěváčková V, Beneš B, Čejchanová M, Šmíd J, Černá M (2006). Blood and urine levels of Pb, Cd and Hg in the general population of the Czech Republic and proposed reference values. Int J Hyg Environ Health.

[CR2] Berglund M, Larsson K, Grandér M, Casteleyn L, Kolossa-Gehring M, Schwedler G, Castaño A, Esteban M, Angerer J, Koch HM, Schindler BK, Schoeters G, Smolders R, Exley K, Sepai O, Blumen L, Horvat M, Knudsen LE, Mørck TA, Joas A, Joas R, Biot P, Aerts D, De Cremer K, Van Overmeire I, Katsonouri A, Hadjipanayis A, Cerna M, Krskova A, Nielsen JKS, Jensen JF, Rudnai P, Kozepesy S, Griffin C, Nesbitt I, Gutleb AC, Fischer ME, Ligocka D, Jakubowski M, Reis MF, Namorado S, Lupsa I-R, Gurzau AE, Halzlova K, Jajcaj M, Mazej D, Tratnik JS, Lopez A, Cañas A, Lehmann A, Crettaz P, Hond ED, Govarts E (2015). Exposure determinants of cadmium in European mothers and their children. Environ Res.

[CR3] Castaño A, Pedraza-Díaz S, Cañas AI, Pérez-Gómez B, Ramos JJ, Bartolomé M, Pärt P, Soto EP, Motas M, Navarro C, Calvo E, Esteban M (2019). Mercury levels in blood, urine and hair in a nation-wide sample of Spanish adults. Sci Total Environ.

[CR4] CDC (2019) Fourth national report on human exposure to environmental chemicals update 866. https://www.cdc.gov/biomonitoring/pdf/fourthreport_updatedtables_feb2015.pdf 20806995

[CR5] Cullen A, Kiberd B, Matthews T, Mayne P, Delves HT, O’Regan M (1998). Antimony in blood and urine of infants. J Clin Pathol.

[CR6] Cullen E, Evans D, Davidson F, Burke P, Burns D, Flanagan A, Griffin C, Kellegher A, Mannion R, Mulcahy M, Ryan M, Biot P, Casteleyn L, Castaño A, Angerer J, Koch H, Esteban M, Schindler B, Navarro C, Kolossa-Gehring M, Fiddicke U, Schoeters G, Hond E, Sepai O, Exley K, Bloemen L, Knudsen L, Joas R, Joas A, Aerts D (2014). Mercury exposure in Ireland: results of the DEMOCOPHES Human Biomonitoring Study. Int J Environ Res Public Health.

[CR7] D’Ovidio F, Rooney JPK, Visser AE, Vermeulen RCH, Veldink JH, Van Den Berg LH, Hardiman O, Logroscino G, Chiò A, Beghi E, For the Euro-MOTOR Group (2017). Critical issues in ALS case-control studies: the case of the Euro-MOTOR study. Amyotrophic Lateral Sclerosis and Frontotemporal Degeneration.

[CR8] Hoet, P., Jacquerye, C., Deumer, G., Lison, D., Haufroid, V., 2013. Reference values and upper reference limits for 26 trace elements in the urine of adults living in Belgium. Clin Chem Lab Med 51. 10.1515/cclm-2012-0688 10.1515/cclm-2012-0688 23314559

[CR9] Jenny-Burri J, Haldimann M, Brüschweiler BJ, Bochud M, Burnier M, Paccaud F, Dudler V (2015). Cadmium body burden of the Swiss population. Food Additives & Contaminants: Part A.

[CR10] Lamkarkach F, Ougier E, Garnier R, Viau C, Kolossa-Gehring M, Lange R, Apel P (2021). Human biomonitoring initiative (HBM4EU): human biomonitoring guidance values (HBM-GVs) derived for cadmium and its compounds. Environ Int.

[CR11] Nisse C, Tagne-Fotso R, Howsam M, Richeval C, Labat L, Leroyer A (2017). Blood and urinary levels of metals and metalloids in the general adult population of Northern France: The IMEPOGE study, 2008–2010. Int J Hyg Environ Health.

[CR12] R Core Team (2022) R: A language and environment for statistical computing. R Foundation for Statistical Computing, Vienna, Austria. URL https://www.R-project.org/

[CR13] Saravanabhavan G, Werry K, Walker M, Haines D, Malowany M, Khoury C (2017). Human biomonitoring reference values for metals and trace elements in blood and urine derived from the Canadian Health Measures Survey 2007–2013. Int J Hyg Environ Health.

[CR14] Schindler BK, Esteban M, Koch HM, Castano A, Koslitz S, Cañas A, Casteleyn L, Kolossa-Gehring M, Schwedler G, Schoeters G, Hond ED, Sepai O, Exley K, Bloemen L, Horvat M, Knudsen LE, Joas A, Joas R, Biot P, Aerts D, Lopez A, Huetos O, Katsonouri A, Maurer-Chronakis K, Kasparova L, Vrbík K, Rudnai P, Naray M, Guignard C, Fischer ME, Ligocka D, Janasik B, Reis MF, Namorado S, Pop C, Dumitrascu I, Halzlova K, Fabianova E, Mazej D, Tratnik JS, Berglund M, Jönsson B, Lehmann A, Crettaz P, Frederiksen H, Nielsen F, McGrath H, Nesbitt I, De Cremer K, Vanermen G, Koppen G, Wilhelm M, Becker K, Angerer J (2014). The European COPHES/DEMOCOPHES project: towards transnational comparability and reliability of human biomonitoring results. Int J Hyg Environ Health.

[CR15] Schulz C, Wilhelm M, Heudorf U, Kolossa-Gehring M (2011). Update of the reference and HBM values derived by the German Human Biomonitoring Commission. Int J Hyg Environ Health.

[CR16] Sun H, Wang D, Zhou Z, Ding Z, Chen X, Xu Y, Huang L, Tang D (2016). Association of cadmium in urine and blood with age in a general population with low environmental exposure. Chemosphere.

[CR17] Taiyun W, Simko V (2021) R package ‘corrplot’: Visualization of a Correlation Matrix (Version 0.92). Available from https://github.com/taiyun/corrplot

[CR18] Vogel N, Murawski A, Schmied-Tobies MIH, Rucic E, Doyle U, Kämpfe A, Höra C, Hildebrand J, Schäfer M, Drexler H, Göen T, Kolossa-Gehring M (2021). Lead, cadmium, mercury, and chromium in urine and blood of children and adolescents in Germany – human biomonitoring results of the German Environmental Survey 2014–2017 (GerES V). Int J Hyg Environ Health.

[CR19] Wickham H (2017). Tidyverse: easily install and load the “tidyverse”. R Package Version.

[CR20] Yoshida K, Bartel A (2021) tableone: Create ‘Table 1’ to Describe Baseline Characteristics with or without Propensity Score Weights. R package version 0.13.0. https://CRAN.R-project.org/package=tableone

