# Supplementary material for: Urine concentrations of selected trace metals in a cohort of Irish adults
Source: Environ Sci Pollut Res Int. 2022 Jun 2;29(50):75356–64. doi: 10.1007/s11356-022-21169-y (PMC9553804; doi:10.1007/s11356-022-21169-y)
Supplement: Supplementary file 1 — Supplementary file1 (DOCX 16 KB) [file 11356_2022_21169_MOESM1_ESM.docx]

Supplementary Table 1. Means and percentiles of urinary toxic metal concentrations by gender in 100 Irish individuals adjusted for creatinine

| Toxic Metal | Sex | N | Arithmetic Mean (μg/g creatinine) | Geometric Mean (μg/g creatinine) | P10% | P25% | P50% | P75% | P90% | P95% |
| --- | --- | --- | --- | --- | --- | --- | --- | --- | --- | --- |
| Aluminium | Female | 42 | 32.3 | 14.4 | 3.9 | 8.2 | 14.3 | 27.1 | 66.8 | 85.6 |
|  | Male | 58 | 24.5 | 12.3 | 2.0 | 6.2 | 11.2 | 30.5 | 63.7 | 103.8 |
| Arsenic | Female | 42 | 49.1 | 17.1 | 2.7 | 6.6 | 17.9 | 42.8 | 153.3 | 194.9 |
|  | Male | 58 | 29.8 | 11.5 | 2.4 | 5.3 | 9.3 | 26.5 | 53.5 | 114.2 |
| Cadmium | Female | 42 | 1.1 | 0.7 | 0.2 | 0.5 | 0.8 | 1.3 | 1.5 | 2.0 |
|  | Male | 58 | 0.5 | 0.4 | 0.0 | 0.3 | 0.5 | 0.7 | 0.9 | 0.9 |
| Chromium | Female | 42 | 1.7 | 1.0 | 0.2 | 0.6 | 1.2 | 2.3 | 3.6 | 4.9 |
|  | Male | 58 | 1.3 | 0.7 | 0.1 | 0.5 | 0.8 | 1.5 | 3.2 | 4.0 |
| Copper | Female | 42 | 15.3 | 9.3 | 1.5 | 7.4 | 9.8 | 15.7 | 24.6 | 43.6 |
|  | Male | 58 | 11.1 | 7.3 | 0.9 | 6.4 | 9.2 | 13.8 | 18.9 | 26.3 |
| Mercury | Female | 42 | 2.9 | 0.6 | 0.1 | 0.3 | 0.5 | 1.0 | 2.8 | 4.7 |
|  | Male | 58 | 1.2 | 0.5 | 0.1 | 0.2 | 0.6 | 1.4 | 2.1 | 3.5 |
| Manganese | Female | 42 | 1.2 | 0.4 | 0.1 | 0.2 | 0.3 | 0.8 | 1.4 | 3.1 |
|  | Male | 58 | 0.6 | 0.3 | 0.1 | 0.2 | 0.3 | 0.6 | 1.4 | 1.7 |
| Lead | Female | 42 | 4.9 | 2.7 | 0.3 | 1.6 | 3.5 | 6.1 | 11.8 | 14.4 |
|  | Male | 58 | 3.7 | 1.9 | 0.2 | 1.3 | 2.1 | 4.5 | 9.6 | 11.5 |
| Selenium | Female | 42 | 34.7 | 23.0 | 3.4 | 22.4 | 27.5 | 34.3 | 38.7 | 77.1 |
|  | Male | 58 | 20.1 | 15.5 | 2.4 | 17.0 | 20.2 | 24.8 | 28.7 | 32.6 |
|  |  |  |  |  |  |  |  |  |  |  |
| Creatinine | Female | 42 | 0.8 | 0.6 | 0.3 | 0.3 | 0.6 | 1.1 | 1.6 | 1.7 |
|  | Male | 58 | 0.9 | 0.7 | 0.2 | 0.4 | 0.8 | 1.4 | 1.5 | 1.7 |
